# Development of word and syllable structure in Chilean children with typical and protracted phonological development

**DOI:** 10.3389/fpsyg.2026.1740877

**Published:** 2026-03-16

**Authors:** Patricio Vergara, Jorge Parada, Yvan Rose, Eliseo Diez-Itza

**Affiliations:** 1 School of Speech-Language Pathology, Austral University of Chile, Puerto Montt, Chile; 2 Institute of Management and Industry, Austral University of Chile, Puerto Montt, Chile; 3 Department of Linguistics, Memorial University of Newfoundland, St. John’s, NL, Canada; 4 Department of Psychology, LOGIN Research Group, University of Oviedo, Oviedo, Spain

**Keywords:** child phonological development, phonological assessment, protracted phonological development, syllable structure, Word Shape Match

## Abstract

The acquisition of syllable structure constitutes a core component of phonological development in early childhood and enables the differentiation of typical and protracted trajectories in Spanish. However, evidence regarding how structural factors (i.e., syllable structure complexity, word length, and within-word position) modulate phonological accuracy remains limited for Chilean Spanish. To address this gap, performance on syllable structure production by children with typical development (TD) was compared with that by children with protracted phonological development (PPD) using the Word Shape Match (WSM) metric. Participants were 160 children aged 3;0–6;11 years, evenly distributed by age and developmental group. Each child completed a Spanish naming task comprising 100 words. Productions were phonetically transcribed and analyzed with Phon 3.1. Results revealed significant differences in WSM scores between TD and PPD across all age ranges, with consistently higher accuracy in the TD group. Independent logistic regression models were applied for each group, with age, word length, syllable type, and syllable position entered as predictors. In both groups, age, word length, and syllable structure complexity emerged as significant predictors; however, in PPD, syllable position also had a significant effect, with higher accuracy in later (medial and final) syllables. WSM thus proved to be a clinically sensitive indicator for distinguishing phonological trajectories, underscoring the importance of integrating suprasegmental measures into the assessment of children’s speech.

## 1 Introduction

Spanish phonological structure, often described as relatively simple, conceals notable areas of structural complexity. Spanish words indeed display a wide range of syllabic patterns, from open CV forms to multisyllabic words with codas or complex onsets. A consideration of this complexity can in turn help us delineate boundaries between typical (TD) and protracted phonological development. Following Stemberger and Bernhardt (2022) and previous work within the framework of Non-linear Phonology, Protracted Phonological Development (PPD) refers to a developmental pattern in which the phonological system is qualitatively similar to that observed in typically developing children, but whose acquisition unfolds over a longer time span. The term PPD is used here as a descriptive construct rather than as a diagnostic label. It allows the characterization of phonological development independently of broader clinical categories such as Speech Sound Disorder or Developmental Language Disorder, while capturing a prolonged developmental course without necessarily presupposing qualitative deviance.

Understanding these divergent trajectories requires outlining the core features of Spanish phonology and how these features influence the acquisition of syllable structure and word shapes.

### 1.1 Phonology of word and syllable structure in Spanish

Words in Spanish may range from very simple structures consisting of a single open syllable (e.g., V: a “to”; CV: no “no”) to complex multisyllabic forms that include closed syllables (CVC: pan “bread”), diphthongs (VV: auto “car”), branched onsets (CCV: plato “dish”; CCVC: trampa “trap”), or combinations of these configurations in words of three, four, or more syllables (e.g., extraordinario “extraordinary”; transportarlo “to transport it”) (Hualde, 2013; Pérez et al., 2025; Diez-Itza and Vergara, in press). In Spanish daily use, and excluding grammatical words, disyllabic words predominate in spontaneous speech (41.98%), followed by monosyllabic (27.7%), trisyllabic (20.3%), tetrasyllabic (6.58%), and pentasyllabic forms (2.12%), with only a very small proportion ( < 1%) of longer words (Quilis, 1993). This strong predominance of disyllabic forms provides a phonotactic and prosodic input that favors early stabilization of simple word shapes and trochaic rhythmic patterns in Spanish-speaking children.

In Spanish (Castilian), 23 syllable types have been identified, of which the most frequent are CV, CVC, V, VC, CCV, CVV, CVVC, and CCVC (Diez-Itza and Vergara, in press; Real Academia Española [RAE], 2011). Among them, the CV structure is considered prototypical due to its high frequency and productivity, whereas syllables with codas (CVC) and complex onsets (CCV) are less common and more restricted (Llisterri, n. d.; Saceda, 2005; Vergara et al., 2023; Vivar, 2009, 2013, 2014; Vivar et al., 2019).

Syllabic organization is also governed by specific constraints: only vowels may serve as syllabic nuclei or function as autonomous syllables; no more than three contiguous vowels may occur in sequence; and both word-initial consonants and word-final consonants are optional (Colina, 2016; Justicia et al., 1996). Consonant clusters (CC) may occur entirely at the beginning of the syllable (CC-; tautosyllabic) or across syllable boundaries (-CC-; heterosyllabic). In addition, prosodic stress in Spanish is distributed within the so-called “three-syllable window” which limits stress placement to one of the final three syllables of the word (Hualde et al., 2001; Hualde, 2013; Pérez et al., 2025).

With respect to syllabic prominence in Spanish, stressed words predominate in the lexicon (63.4%), although unstressed lexical items also occur with a substantial frequency (36.6%). Within stressed words, final stress accounts for 17.7% of cases, whereas penultimate stress is overwhelmingly predominant (79.5%) (Quilis, 1983). Consequently, in disyllabic words, trochaic stress patterns (Sw) are markedly more frequent than iambic patterns (wS), although the rhythm of speech is isosyllabic and not marked by stress but by syllable duration (Diez-Itza and Vergara, in press; Pérez et al., in press).

Although detailed corpus-based distributions of syllable types as a function of prosodic prominence (stress position or word-final position) are not currently available for Chilean Spanish, previous work has shown that prominence effects may modulate phonological development when frequency alone does not fully account for acquisition patterns (Ben-David and Bat-El, 2017; Cilibrasi et al., 2015; Echols and Newport, 1992). In this sense, syllable position within the word constitutes an indirect but robust proxy for prosodic salience, as word-final and stressed syllables often concentrate greater perceptual and articulatory stability. Vergara et al. (2021) showed that tautosyllabic consonant clusters are produced with higher accuracy when they occur in stressed syllables, particularly in children with protracted phonological development, indicating that prosodic prominence can facilitate phonological accuracy beyond frequency of occurrence.

In Chilean Spanish, it is particularly relevant to consider dialectal phenomena that affect the realization of syllable shapes. One of the most well-documented is the aspiration of /s/ in coda position, which results in structures such as CVC being phonetically realized as [CVh] or [CVs^h^]. Although these realizations do not alter the target syllable type (CVC), they reduce the phonetic stability and perceptual salience of codas, increasing variability in children’s speech production (Cerda et al., 2015).

### 1.2 Acquisition of word and syllable structure in TD and PPD

The acquisition of word phonology follows a trajectory of increasing complexity. In early stages, simplification appears through reduced word length and omission of marginal segments (Bosch, 2004; Coloma et al., 2010; Ingram, 1989; Pavez et al., 2013). Children between 1 and 2 years rarely produce words longer than two syllables; around 3 years they begin to produce trisyllabic forms, and by five, tetrasyllabic and pentasyllabic words become consistent (Miras, 1992). This progression is particularly evident in the acquisition of polysyllabic words (James et al., 2002).

James et al. (2008) propose a five-stage model for the acquisition of polysyllabic words, ranging from fidelity to the stressed syllable and word duration (1;0–2;3), through preservation of syllable number (2;4–3;11), to phoneme matching (4;0–6;11), rhythmic accuracy (7;0–10;11), and adult-like production (11;0 onwards). Development advances through stronger faithfulness and weaker markedness constraints (Barlow and Gierut, 1999). After reviewing 53 studies, Masso et al. (2018) expanded this model into the Framework of Polysyllable Maturity, confirming that words with three or more syllables are sensitive indicators of phonological maturity and potential predictors of later literacy difficulties. This complexity rests on the gradual mastery of the syllabic structures composing the word.

A major contribution to this perspective is the special issue edited by Demuth (2006) on the acquisition of prosodic word structures, which brought together cross-linguistic evidence (Catalan: Prieto, 2006; European Portuguese: Vigário et al., 2006; Japanese: Ota, 2006; Spanish: Lleó, 2006; and English: Demuth et al., 2006), showing that children’s early word productions are systematically shaped by the prosodic and syllabic organization of their target language. These studies demonstrated that phonological development cannot be reduced to the acquisition of individual segments but rather involves the progressive construction of prosodic word forms, including syllable structure, stress patterns, and rhythmic organization. This work provided strong empirical support for viewing the word as a hierarchical phonological unit and highlighted the central role of prosodic structure in explaining both typical and atypical developmental trajectories.

In Spanish, Serra et al. (2000) found that between 1;6 and 4;3, children first master syllables with nuclei (V, VV) and simple CV structures, later producing forms with complex onsets (CCV), diphthongs, and codas (VC, CVC, CCVC). Tapia (2003) described four milestones: (1) simple forms (V, CV); (2) codas (VC, CVC); (3) complex onsets (CCV, CCVC); and (4) highly complex forms (VCC, CVCC, CCVCC). The first three emerge during preschool, the last consolidates in primary school. Saceda (2005) further observed that onsetless syllables (V, VC) and diphthongs (VV) appear before codas. These stages show parallels, at a general prosodic level, with those described for English and Dutch by Demuth and Fee (1995), who outlined four developmental phases: simple unmarked forms, first binary words, incorporation of stress within a single foot, and consolidation of multi-foot prosodic words. However, the specific syllabic structures and phonotactic constraints that instantiate these phases differ across languages and are determined by the phonological system of each language.

In Spanish–Catalan bilinguals, Aguilar-Mediavilla et al. (2002) analyzed the phonology of children with Specific Language Impairment and Language Delay from a larger longitudinal study at the initial testing age of 3;10 (range: 3;6–4;01), and reported delayed acquisition of segments and syllabic structures in children with specific language impairment, marked by persistent simplifications. Later, Aguilar-Mediavilla et al. (2020) confirmed that 6-year-old children with developmental language disorder show reduced accuracy for whole words, syllables, and phonemes, evidencing fundamentally phonological rather than articulatory difficulties.

Complementing these group findings, a detailed case study of a 5-year-old Spanish-speaking child with protracted phonological development demonstrated that, despite a near-complete consonant and vowel inventory, production accuracy was severely constrained at the word level, with low Whole Word Match, simplification of multisyllabic structures, deletion of unstressed syllables, and marked limitations on consonant and vowel sequences, highlighting the central role of word structure and complexity in atypical development (Vergara et al., 2022). This profile is consistent with cross-linguistic evidence within the special issue “Individual profiles in protracted phonological development across languages,” which shows that children with PPD across diverse languages exhibit persistent constraints on word length, syllable structure, stress patterns, and structure–segment interactions, underscoring word shape development as a core locus of difficulty in atypical phonological development (Stemberger and Bernhardt, 2022).

### 1.3 The contribution of non-linear phonology to studies on Spanish acquisition

Non-linear Phonology integrates evidence on phonological development into a hierarchical model encompassing the segmental, syllabic, foot, and prosodic word levels. This framework enables the use of suprasegmental metrics such as Word Shape Match (WSM), which evaluate correspondence between a child’s production and the adult target. Whereas traditional accuracy measures, such as Percentage of Consonants Correct (PCC), focus on segmental precision, WSM captures the organization of syllabic and prosodic structure at the word level, providing a more comprehensive information about phonological development. Although widely supported in Clinical Phonology (Vihman, 2016; Ingram and Ingram, 2001) and developed in the cross-linguistic project led by Bernhardt and Stemberger (University of British Columbia^1^), its application to Spanish remains limited.

From a clinical perspective, Non-linear Phonology was developed in response to the limitations of process-based approaches derived from Natural Phonology. As argued by Bernhardt and Stoel-Gammon (1994), phonological process analyses focus primarily on describing surface mismatches between adult targets and child productions, but they do not capture entirely how segments are organized within syllables and prosodic structures. In contrast, Non-linear Phonology provides a representational framework that integrates segmental, syllabic, and prosodic levels, allowing clinicians to identify whether difficulties arise from segmental contrasts, syllable structure, or their interaction (Stemberger and Bernhardt, 2022).

Within Spanish-speaking contexts, Bernhardt et al. (2015) distinguished TD and PPD in Granada preschoolers (92%–95% vs. 63%–75% WSM), and Pérez et al. (2019) reported comparable findings in Chilean children with PPD (mean = 73.4%). These studies highlight WSM as a sensitive indicator of phonological development, though research in Spanish is still scarce and restricted to small samples. Integrating WSM with detailed syllabic characterization may thus offer a clinically relevant framework for differentiating TD and PPD trajectories. A more recent study analyzed complexity and sequence constraints, from perspective of constraint-based non-linear phonology, in a Granada Spanish-speaking 4-year-old with protracted phonological development and found asynchronies in acquisition between segmental and syllabic levels (Vergara et al., 2022).

These findings in Spanish, showing that word-level and syllable-level measures derived from Non-linear Phonology are sensitive to phonological differences between TD and PPD, are consistent with evidence from other languages, including English (Chung et al., 2022), Icelandic (Másdóttir and Bernhardt, 2022), French (Bérubé et al., 2020; Bérubé and Spoor, 2022), Swedish (Lundeborg Hammarström, 2018), and Arabic (Ayyad et al., 2016).

The present study therefore aimed to describe and compare the acquisition of word and syllable structure in children with TD and PPD using the WSM metric, examining the effects of age, syllable type, word length, and syllable position on phonological accuracy.

## 2 Methods

### 2.1 Participants

The sample consisted of 160 monolingual Chilean Spanish-speaking children, divided into two main groups: TD and PPD. Each group was organized into eight age bands, yielding a total of 16 subgroups balanced by sex (5♀/♂5), covering the range from 3;0 to 6;11.

The TD group (*n* = 80) included participants with no history of language impairment, all enrolled in mainstream educational settings. Their age distribution was as follows: 3;0–3;5 years (*M* = 3;4, SD = 1.1 months), 3;6–3;11 (*M* = 3;9, SD = 1.3 months), 4;0–4;5 (*M* = 4;2, SD = 1.9 months), 4;6–4;11 (*M* = 4;8, SD = 2.2 months), 5;0–5;5 (*M* = 5;4, SD = 1.9 months), 5;6–5;11 (*M* = 5;9, SD = 3.1 months), 6;0–6;5 (*M* = 6;2, SD = 1.8 months), and 6;6–6;11 (*M* = 6;8, SD = 1.5 months).

The PPD group (*n* = 80) included children who obtained scores ≥ +2 SDs on a standardized phonology test for the Chilean population [*Test para Evaluar Procesos Fonológicos de Simplificación, versión Revisada* (Test for Evaluating Phonological Simplification Processes, Revised version), TEPROSIF-R; Pavez et al., 2008]. They had been previously diagnosed with developmental language disorder (DLD), through national assessment programs, including evaluation of grammatical difficulties with the *Test Exploratorio de Gramática Española*, Chilean adaptation (Exploratory Test of Spanish Grammar) (STSG; Pavez, 2014). Children with hearing impairments, structural anomalies of the speech organs, or diagnoses of neurodevelopmental disorders other than DLD were excluded, based on a comprehensive assessment battery including the following instruments: *Test para Evaluar Procesos Fonológicos de Simplificación, versión Revisada* (TEPROSIF-R; Pavez et al., 2008), *Test de Comprensión Auditiva del Lenguaje de E. Carrow*, Chilean adaptation (Test of Auditory Comprehension of Language) (TECAL; Pavez, 2004), *Test Exploratorio de Gramática Española*, Chilean adaptation (STSG; Pavez, 2014), the *Pragmatic Protocol* (Prutting and Kirchner, 1987), the *Articulation Repetition Test* (TAR; Schwalm, 1981), and an *Observation Checklist of Speech Articulatory Organs* (Busto, 1995). Furthermore, the parents were asked to provide information about the developmental and clinical records of the children through a structured interview.

The participants with PPD attended either specialized language disorder schools or mainstream schools with School Integration Programs (Chilean Ministry of Education, 2009). Their age distribution was as follows: 3;0–3;5 years (*M* = 3;3, SD = 1.5 months), 3;6–3;11 (*M* = 3;8, SD = 1.8 months), 4;0–4;5 (*M* = 4;3, SD = 1.7 months), 4;6–4;11 (*M* = 4;8, SD = 1.5 months), 5;0–5;5 (*M* = 5;3, SD = 2.2 months), 5;6–5;11 (*M* = 5;8, SD = 1.8 months), 6;0–6;5 (*M* = 6;2, SD = 1.9 months), and 6;6–6;11 (*M* = 6;8, SD = 2.4 months).

All participants came from middle socio-economic status families and lived in urban areas of central-southern Chile. Informed consent was obtained from parents or guardians, along with child assent and relevant medical background information. The study protocol was approved by the Subcommittee on Bioethics in Human Research of Austral University of Chile, under FONDECYT Project No. 11230656.

### 2.2 Materials and procedures

The Spanish Phonology Test (PFE; Bernhardt et al., 2016) was administered to elicit the words for phonological analysis. The test can be applied digitally (via computer or tablet) or through a booklet containing 100 printed picture plates representing nouns and verbs of varying length (9 monosyllables, 56 disyllables, 25 trisyllables, 9 four-syllable words, and 1 five-syllable word). The items also vary in stress pattern. Excluding monosyllabic words, which do not display contrastive stress placement in Spanish, the remaining items include 5 proparoxytones, 79 paroxytones, and 7 oxytones. In addition, the items vary in syllabic structure (24 items include tautosyllabic consonant clusters and another 24 contain diphthongs). The consonantal codas represented in the materials include different phonological classes, such as nasals and liquids, as well as the fricative /s/, which is the only fricative occurring in coda position in the test items. Twenty-three items contained diphthongs. Of these, 10 included falling diphthongs (/ai, ei, oi, au/) and 14 included rising diphthongs (/ia, ie, io, ua, ue/), with one item containing both types of diphthongs within the same word. These design features ensure adequate segmental coverage, so that each phoneme is elicited at least twice across the test items.

Administration of the PFE was carried out individually in a quiet room by a qualified speech and language therapist. All sessions were sound-recorded in WAV using a TASCAM DR-05X recorder (44.1 kHz/16-bit) with omnidirectional stereo condenser microphones placed on the table approximately 30–40 cm from the child’s mouth. Following the test instructions, each participant was first familiarized with a few common images used as examples; subsequently, the 100 target words were elicited from the picture plates. If a child did not spontaneously produce a word upon seeing the picture, a choice between two alternatives was offered (delayed imitation), and if difficulty persisted, direct repetition was requested. In over 95% of cases, words were produced spontaneously.

The responses were transcribed phonetically (broad phonetic transcription) and entered in the Phon 3.1 software (Hedlund and Rose, 2020). For each participant, an independent Phon corpus was created, including the audio file, the orthographic transcription of the target words, and broad phonetic transcriptions of both the target form and the child original production using the International Phonetic Alphabet (International Phonetic Association [IPA], 1999). The initial word phonetic transcriptions were independently conducted by two specialists, achieving an agreement rate of 99.5%. Any discrepancies were subsequently reviewed and resolved by the principal investigator.

Some target words allow variable syllabic parsing in Spanish (e.g., *hueso*). Syllable structure was determined based on the child’s phonetic production, using broad phonetic transcription. As mentioned above, socio-dialectal variants of Chilean Spanish may also include aspiration of coda /s/. All these cases were treated as acceptable realizations and were not coded as errors.

### 2.3 Data analysis

Word Shape Match scores were calculated for each participant using Phon 3.1 (Hedlund and Rose, 2020). When the child’s production matched the adult target in terms of syllabic structure, a value of 1 was automatically assigned by Phon [e.g., /’pero/ “dog” → adult target (CV.CV), child production (CV.CV)]; otherwise, a value of 0 was assigned. The output of Phon also provided percentage scores of WSM.

Mean WSM scores between groups were compared using Student’s *t*-tests. When the assumption of homoscedasticity was violated, Welch’s correction was applied; in cases of severe deviations from normality, equivalent non-parametric tests (Mann–Whitney) were used. The magnitude of group differences was reported using Cohen’s *d* as a measure of effect size.

Complementarily, an analysis of the syllable types present in each word was conducted using PhonShell scripts^2^. The analysis used in the current study will soon be made available to all the research community through an upcoming release of Phon 4. Using this analysis, we evaluated nine distinct syllable types: CV, V, VV, CVC, VC, CCV, CCVC, CVVC, and CVV. These categories ranged from simple and frequent forms in Spanish, such as CV syllables, to more complex structures involving codas, diphthongs, or consonant clusters. This allowed for the examination of children’s phonological accuracy considering not only overall WSM performance but also variation as a function of syllable structure complexity. As with WSM, when the child’s production matched the target syllabic structure, a value of 1 was assigned; otherwise, a value of 0. Subsequently, mean differences were tested (Student’s t, Welch’s t, or Mann–Whitney, as appropriate) to compare TD and PPD groups for each syllable type.

The analysis of factors modulating the probability of correct syllable production was carried out using a binary logistic regression model, a technique suitable for dichotomous dependent variables. This approach estimates the probability of the event of interest, accurate syllable production (1 = correct, 0 = incorrect), based on a set of linguistic and developmental predictors, expressing relationships in terms of the logarithm of the odds ratios (log-odds). Interpretative parameters included estimated coefficients (β), standard errors, significance values (*p*), and odds ratios (OR), which quantify the magnitude and direction of the effects (Hosmer and Lemeshow, 2000). The dependent variable corresponded to syllable accuracy, while independent variables included participant age (in 6-month intervals), syllabic structure, position within the word, and total number of syllables. The analyses were conducted at the token level, with each syllable production treated as an individual observation within the logistic regression models.

Word length (monosyllabic–pentasylabic), syllable position within the word (1st–5th syllable, determined according to the temporal production sequence), and age band (organized in equal 6-month intervals between 3;0 and 6;11 years: 3;0–3;5, 3;6–3;11, 4;0–4;5, 4;6–4;11, 5;0–5;5, 5;6–5;11, 6;0–6;5, and 6;6–6;11) were treated as ordinal quantitative variables, representing hierarchical progressions linked to structural complexity and developmental maturation of speech. In contrast, syllable type was treated as a nominal qualitative variable, defined according to phonotactic configuration (e.g., CV, CVC, CCV, etc.). The CCV structure was selected as the reference category because it corresponds to one of the latest-acquired forms in Spanish child phonology and yields odds ratios greater than one, thus allowing a more direct and consistent interpretation of the relative effects of the remaining categories on the probability of correct production.

All analyses were performed in the R environment (R Core Team, 2022), using the stats package and the *glm* function for binary logistic regression estimation. The full list of words included in the PFE, along with their phonetic transcription, structure, and syllabic composition, is provided in the Supplementary Table 1.

## 3 Results

### 3.1 Word Shape Match in TD and PPD

Table 1 presents the percentage scores obtained in the WSM metric and the comparison of means between children with TD and PPD across different age bands. In all age ranges analyzed, a significant difference was observed between the two groups, with consistently higher values in the TD group. Descriptive statistics for WWM, PCC, and CV Match for each age group and developmental group are provided in Supplementary Table 2.

**TABLE 1:** Word Shape Match (WSM) mean differences in children with TD and PPD across age bands.

Age band	TD WSM % (SD)	PPD WSM % (SD)	Statistical test	*P*-value	Cohen’s *d*
3;0–3;5	88 (4)	73 (6)	*t* = 6.71	<0.001	3.00
3;6–3;11	93 (6)	77 (10)	*t* = 4.58	<0.001	2.05
4;0–4;5	93 (4)	78 (7)	*t* = 5.70	<0.001	2.55
4;6–4;11	98 (1)	79 (11)	w = 100	<0.001	3.62
5;0–5;5	99 (1)	85 (11)	tw = 3.88	0.003	1.74
5;6–5;11	98 (3)	85 (10)	w = 97	<0.001	2.62
6;0–6;5	99 (1)	87 (7)	tw = 5.75	<0.001	2.57
6;6–6;11	99 (0)	91 (4)	w = 100	<0.001	3.62

TD, typical development; PPD, protracted phonological development; t, Student’s t statistic; tw, Welch’s t statistic; w, Wilcoxon statistic; Cohen’s d = effect size.

From the age of five onwards, the TD group reached near-ceiling levels (≥0.98), maintaining stable performance up to age 6;11. In contrast, children with PPD showed slower progress and relatively stable performance, ranging between 0.73 and 0.91, without reaching the levels achieved by the TD group.

Effect sizes (Cohen’s *d*) further reinforced the magnitude of this gap: in the initial age bands (3;0–4;11), very large effects were observed (*d* = 2.0–3.6), indicating robust differences from early stages. Although in the older age bands (5;0–6;11) effect sizes decreased slightly (*d* = 1.7–2.6), they remained within the range of large effects, confirming that the discrepancy between TD and PPD persists and becomes particularly evident in more complex word structures from age 5;0 onwards.

### 3.2 Accuracy by syllable structure in TD and PPD

Table 2 presents the mean observed accuracy values (based on Phon dichotomous analyses: 0 incorrect–1 correct) for each type of syllable structure, comparing children with TD and PPD across different age bands. Overall, children with TD showed steady progress throughout development, reaching near-ceiling values (≥0.95) from age 5;0, whereas children with PPD displayed lower and more variable performance, ranging between 0.06 and 0.98 depending on age and syllable type.

**TABLE 2:** Mean observed accuracy values by syllable type, developmental group (TD and PPD), and age band.

Group	Syllable	3;0–3;5	3;6–3;11	4;0–4;5	4;6–4;11	5;0–5;5	5;6–5;11	6;0–6;5	6;6–6;11
TD	CCV	0.40	0.59	0.61	0.91	0.90	0.85	0.94	0.97
	CCVC	0.45	0.64	0.58	0.83	0.97	0.85	0.97	0.95
	CVVC	0.56	0.72	0.74	0.94	0.98	1.00	1.00	0.96
	CVV	0.78	0.95	0.96	0.98	0.97	0.98	0.97	0.99
	VC	0.73	0.79	0.87	1.00	1.00	0.90	1.00	1.00
	CVC	0.88	0.94	0.96	0.98	0.99	0.99	0.99	0.99
	VV	0.93	1.00	0.93	0.93	0.95	0.97	1.00	1.00
	V	0.96	0.99	0.98	1.00	0.99	1.00	1.00	1.00
	CV	0.98	0.92	0.98	0.99	1.00	1.00	1.00	1.00
PPD	CCV	0.06**	0.21**	0.13**	0.34***	0.35**	0.38***	0.35***	0.61***
	CCVC	0.06**	0.23*	0.11**	0.34**	0.45**	0.43*	0.43***	0.60**
	CVVC	0.12***	0.18**	0.40**	0.40**	0.60***	0.49***	0.61***	0.71**
	CVV	0.31***	0.43***	0.57**	0.50***	0.73*	0.68*	0.77**	0.82***
	VC	0.45	0.50	0.62	0.31***	0.71*	0.56*	0.75**	0.78**
	CVC	0.59***	0.58**	0.71**	0.68***	0.82***	0.78**	0.84**	0.87**
	VV	0.55**	0.60**	0.79	0.69*	0.80	0.83*	0.90	0.95
	V	0.86*	0.92	0.89	0.82**	0.96	0.93*	0.94*	0.99
	CV	0.92**	0.95**	0.93**	0.94***	0.96***	0.96**	0.97***	0.98***

TD, typical development; PPD, protracted phonological development.

***p < 0.001;

**p < 0.01;

*p < 0.05. Statistical comparisons were conducted between TD and PPD groups within each age band and syllable type. Reported *p*-values correspond to between-group contrasts for each syllable structure across developmental stages.

The pattern was particularly evident in complex syllabic structures (CCV, CCVC, CVVC, CVV, CVC), where differences between groups were consistent and statistically significant across all age bands. In these syllables, children with TD achieved high levels of accuracy much earlier, whereas those with PPD progressed more slowly generating a growing gap. In contrast, in CV syllables the children with PPD presented very high accuracies from age 3;0, but statistical differences persisted at all ages as the TD group presented near-ceiling or ceiling accuracies.

### 3.3 Syllable accuracy predictors

#### 3.3.1 Logistic regression model for the TD group

The logistic regression model applied to the TD group showed that syllable accuracy was significantly modulated by age, word length (number of syllables), and syllable structure, whereas syllable position within the word did not present a significant effect, as we can see in Table 3.

**TABLE 3:** Logistic regression model for the typical development (TD) group.

Factor	β coefficient	Standard error	Statistic	*P*-value	Odds ratio
Intercept	0.22	0.16	1.36	0.174	–
Age band	0.50	0.02	21.93	<0.001***	1.65
Number of syllables	−0.39	0.06	−6.16	<0.001***	0.67
Syllable position	−0.01	0.07	−0.12	0.901	0.99
**Syllable type**					
CCVC	−0.18	0.14	−1.22	0.221	0.84
CV	4.04	0.14	29.90	<0.001***	56.96
CVC	2.34	0.13	17.65	<0.001***	10.41
CVV	2.01	0.16	12.88	<0.001***	7.45
CVVC	0.70	0.17	4.10	<0.001***	2.01
V	4.01	0.39	10.16	<0.001***	55.20
VC	1.67	0.27	6.12	<0.001***	5.30
VV	2.03	0.31	6.56	<0.001***	7.62

Syllable type reference category = CCV;

***p < 0.001.

The age factor showed a highly significant effect, with an OR indicating that syllable accuracy increased by a factor of 1.65. This finding confirms a developmental effect of chronological age on the consolidation of the phonological system. Word length had a significant negative effect, indicating that longer words reduced syllable accuracy.

Regarding syllable type, all structures, except CCVC, predicted syllable accuracy when taking CCV as the reference category. The simplest structures, CV and V, were the best predictors of accuracy, which indicates that they are the easiest to produce; on the other hand, CVVC and VC present the lowest ORs. This gradient of complexity reflects the direct influence of phonotactic structure on phonological accuracy.

Taken together, the model indicates that age and syllable structure complexity are the strongest predictors of phonological performance in children with typical development. As age increases, there is a systematic improvement in accuracy, particularly in simpler syllable structures, suggesting progressive maturation of both phonetic control and syllabic planning.

#### 3.3.2 Logistic regression model for the PPD group

The logistic regression model applied to the PPD group revealed significant effects of age, syllable position, syllable structure, and word length on the probability of accurate syllable production (see Table 4). The age factor was highly significant, indicating a progression of phonological accuracy, although of smaller magnitude than that observed in the TD group. Contrasting with the TD group, syllable position had a significant effect in the PPD group, as syllables occurring in later positions within the word were more likely to be produced correctly.

**TABLE 4:** Logistic regression model for the protracted phonological development (PPD) group.

Factor	β coefficient	Standard error	Statistic	*P*-value	Odds ratio
Intercept	−1.02	0.10	−9.81	<0.001***	–
Age band	0.26	0.01	23.45	<0.001***	1.30
Number of syllables	−0.65	0.04	−17.71	<0.001***	0.52
Syllable position	0.29	0.03	8.37	<0.001***	1.33
**Syllable type**					
CCVC	−0.12	0.12	−0.95	0.344	0.89
CV	4.31	0.08	52.39	<0.001***	74.48
CVC	2.09	0.08	25.90	<0.001***	8.09
CVV	1.44	0.09	16.59	<0.001***	4.23
CVVC	0.65	0.13	5.20	<0.001***	1.92
V	3.94	0.16	24.65	<0.001***	51.49
VC	2.03	0.21	9.75	<0.001***	7.59
VV	1.99	0.15	13.25	<0.001***	7.31

Syllable type reference category = CCV;

***p < 0.001.

Word length had a significant negative effect, indicating that for each additional syllable, the probability of a correct production was reduced by approximately a half. This finding confirms that word length imposes an additional phonological load, affecting children with PPD more markedly.

Regarding syllable structure, taking again CCV as the reference category, all other structures, except CCVC, predicted accurate production. The simplest structures, CV and V, were even better predictors of accuracy than in the TD group, which indicates that proportionally CCV is more difficult for these children, on the other hand, CVVC and CVV present the lowest ORs.

Taken together, the results of the model indicate that, in PPD, syllable production is modulated by both linguistic and positional factors, showing a positive developmental effect of age but also a strong sensitivity to structural complexity and word length.

## 4 Discussion

The results of this study confirm asynchronous phonological trajectories in Chilean children with TD and PPD between the ages 3–7, in terms of accuracy in both the overall structure of words and syllabic structures. Children with TD started with high percentages in WSM (88) at age 3 and reached near ceiling percentages (>98) by age 4;6, whereas those with PPD started with lower percentages (73) and, with a less steep slope in their trajectory, maintained a protracted development that was far from being completed by age 7. This differences, supported by large effect sizes across all age groups, reflects phonological asynchronies at segmental, suprasegmental, and prosodic levels. Clinically, the effect of age reflects differentiated developmental trajectories rather than simple maturation. While phonological accuracy increases with age in both groups, children with PPD do not reach optimal levels of performance, highlighting the need for specialized intervention to support the consolidation of more complex phonological structures. Different rates of development may indicate distinct pathways, as it has been suggested: asynchronous means atypical (Diez-Itza et al., 2021; Levy and Eilam, 2013).

The WSM measure then captures word and syllabic structure beyond the TEPROSIF-R test previously administered to the participants. While the TEPROSIF-R and WSM are both sensitive to phonological development, as they both analyze segmental substitutions and omissions, they target different levels of representation. The TEPROSIF-R focuses on segmental and process-based patterns of error, whereas WSM captures the child’s ability to preserve the global prosodic and structural shape of words.

A detailed analysis of syllable accuracy by types of syllables demonstrated that WSM is not homogeneous but varies depending on the types of syllables present in each word. For both groups, the more complex syllables showed lower accuracy, but in the case of the PPD group, the levels were much lower and increased less with age. In less complex syllables, especially CV, the level of accuracy in children with PPD was high from age 3;0 (syllable match = 0.92), but differences persisted because the group with TD performed at near ceiling levels (syllable match = 0.98), although the magnitude of the gap was much smaller in clinical terms. The fact that CV syllables also differentiate TD and PPD is theoretically relevant, as CV is typically regarded as an unmarked and early-acquired structure. This indicates that PPD affects not only complex syllabic configurations but also the stability of basic phonological representations. The fact that there were no differences when producing vowel segments or diphthongs confirms that vowel elements are the earliest acquired by participants with PPD.

These findings are consistent with research documenting similar divergences in typical and atypical trajectories. Bernhardt et al. (2015) reported that WSM distinguished Granada preschoolers with TD and PPD from age 4, while Pérez et al. (2019) found scores closely matching those observed here (around 73%) in Chilean children with PPD aged 3;0–3;11. Likewise, the results align with Aguilar-Mediavilla et al. (2002, 2020), who indicated that phonological difficulties in developmental language disorder are manifest as persistent simplifications and reduced stability of complex syllabic combinations, a phenomenon observed in CCV, CCVC, and CVVC structures.

From a Non-Linear Phonology perspective, Bernhardt et al. (2020) also highlighted that PPD reflects interactions across multiple representational levels, where word and syllable structures play a key modulatory role. Consistent with this view, Bérubé et al. (2020) demonstrated that increasing word length and syllable structure complexity systematically reduce phonological accuracy, underscoring that linguistic structure and processing load jointly shape children’s phonological performance.

In a previous study, centered on constraint-based Non-Linear Phonology analyses in Granada Spanish, Vergara et al. (2022) reported that a 4-year-old child with PPD showed markedly lower WWM and WSM scores, particularly in trisyllabic and structurally complex words. Their analysis demonstrated that increasing word length and syllable structure complexity imposed strong organizational and sequencing constraints on production accuracy, paralleling the present data and reinforcing the interpretation of word structure as a key modulator of phonological performance.

Logistic regression analyses provided quantitative evidence of factors modulating syllabic accuracy. In both groups, age, word length, and syllable structure were predictors of phonological accuracy, whereas in PPD, syllable position also had a significant predictive effect. Syllables in later positions tend to be produced more accurately, suggesting that earlier positions are marked (i.e., more complex and later acquired). The finding that later syllables are produced more accurately in children with PPD is compatible with previous evidence showing that prosodic prominence, including stress and word-final position, facilitates phonological stability (Echols and Newport, 1992; Ben-David and Bat-El, 2017; Cilibrasi et al., 2015). This suggests that positional effects may partially compensate for reduced stability of phonological representations in children with PPD. Although formal implicational relationships could not be deduced as complex mediating factors could be involved (Watts and Rose, 2020).

Developmentally, the progression confirms the hierarchical patterns described in TD by Tapia (2003), Saceda (2005): advancement from simple syllables (V, CV) toward more complex configurations (CVC, CCV, CCVC). Furthermore, results of the present research are consistent with studies of late phonological development in TD children showing non-linear trajectories and phonological stages that are by age 6 at their final time (Diez-Itza et al., 2001; Diez-Itza and Martínez, 2004).

The present study was exploratory thus subject to limitations and needing further research and analyses. Although the sample size proved to be adequate for the objectives, extending representation across age bands would strengthen the validity of the findings. Stress was not included as an independent factor in the present analyses because the PFE was not designed to manipulate stress position systematically, and because the strong predominance of paroxytone patterns in Spanish limits its variability as a predictor. Future research should explicitly address stress as a controlled variable to further explore its interaction with syllable structure and phonological accuracy; it should also consider other dimensions of syllabic production and include complementary indicators, such as Whole Word Match or Percentage of Consonants Correct, to expand multidimensional analysis of PPD in Chilean Spanish. The generalized linear model used in the analysis (logistic regression) is limited to fixed effects thus it fails to give an account of random effects due to nested observations within lexical items. Future research should extend the analysis to a generalized linear mixed model (mixed effects logistic regression).

In conclusion, the results of the present study indicate that PPD represents a distinct phonological trajectory characterized by a more linear, less pronounced, longer and asynchronous gradient of development, with reduced phonological generalization, and reliance on simpler phonotactics. Accuracy is especially lower in complex syllabic structures. From the Non-Linear Phonology perspective, the results emphasize the view of the word as a hierarchical unit integrating segments, syllables, and prosodic feet. The percentage of WSM, by evaluating global structure accuracy, captures the child’s level of suprasegmental organization and complements segmental metrics, refining the clinical characterization of phonological development. A multidimensional analysis may provide a more integrated view of phonological development and may enhance early identification of asynchronous atypical trajectories. In clinical practice, the combined use of suprasegmental metrics such as WSM and detailed structural analyses could optimize early detection and inform intervention strategies focused on expanding syllable structure complexity and improving prosodic stability.

## Data Availability

The original contributions presented in this study are included in this article/Supplementary material, further inquiries can be directed to the corresponding author.

## Footnotes

1
https://phonodevelopment.sites.olt.ubc.ca

2
https://www.phon.ca/phon-manual/tools/phonshell.html
