# Supplementary material for: Development of word and syllable structure in Chilean children with typical and protracted phonological development
Source: Front Psychol. 2026 Mar 16;17:1740877. doi: 10.3389/fpsyg.2026.1740877 (PMC13033755; doi:10.3389/fpsyg.2026.1740877)
Supplement: Supplementary file 1 [file Table_1.docx]

Supplementary Material

**Supplementary Table 1.**

**Table S1***List of words from the Spanish Phonology Test, their structure, and syllabic composition*

| **No.** | **Orthographic word** | **English translation** | **IPA target** | **Word structure** | **No. of syllables** | **Syllable types** |
| --- | --- | --- | --- | --- | --- | --- |
| 1 | perro | dog | /ˈpero/ | CVCV | 2 | CV.CV |
| 2 | hueso | bone | /ˈ{g}weso/ | (C)VVCV | 2 | (C)VV.CV |
| 3 | gato | cat | /ˈgat̪o/ | CVCV | 2 | CV.CV |
| 4 | blanco | white | /ˈblaŋko/ | CCVCCV | 2 | CCV.CV |
| 5 | casa | house | /ˈkasa/ | CVCV | 2 | CV.CV |
| 6 | llave | key | /ˈʤaβe/ | CVCV | 2 | CV.CV |
| 7 | techo | roof | /ˈt̪eʧo/ | CVCV | 2 | CV.CV |
| 8 | chimenea | chimney | /ʧimeˈnea/ | CVCVCVV | 4 | CV.CV.CV.V |
| 9 | escalera | staircase | /e{s}kaˈleɾa/ | V(C)CVCVCV | 4 | V(C).CV.CV.CV |
| 10 | mesa | table | /ˈmesa/ | CVCV | 2 | CV.CV |
| 11 | silla | chair | /ˈsiʝa/ | CVCV | 2 | CV.CV |
| 12 | teléfono | phone | /t̪eˈlefono/ | CVCVCVCV | 4 | CV.CV.CV.CV |
| 13 | lámpara | lamp | /ˈlampaɾa/ | CVCCVCV | 3 | CVC.CV.CV |
| 14 | luz | light | /ˈlu{s}/ | CV(C) | 1 | CV(C) |
| 15 | cuadro | picture | /ˈkwad̪ɾo/ | CVVCCV | 2 | CVV.CCV |
| 16 | baño | bathroom | /ˈbaɲo/ | CVCV | 2 | CV.CV |
| 17 | fuego | fire | /ˈfweɣo/ | CVVCV | 2 | CVV.CV |
| 18 | abierta | open | /aˈβjeɾt̪a/ | VCVVCCV | 3 | V.CVVC.CV |
| 19 | aire | air | /ˈai̯ɾe/ | VVCV | 2 | VC.CV |
| 20 | azul | blue | /aˈsul/ | VCVC | 2 | V.CVC |
| 21 | jamón | ham | /xaˈmon/ | CVCVC | 2 | CV.CVC |
| 22 | pescado | fish | /pe{s}ˈkaðo/ | CV(C)CVCV | 3 | CV(C).CV.CV |
| 23 | uvas | grapes | /ˈuβa{s}/ | VCV(C) | 2 | V.CV(C) |
| 24 | fruta(s) | fruit | /ˈfɾut̪a{s}/ | CCVCV(C) | 2 | CCV.CV(C) |
| 25 | fresa | strawberry | /ˈfɾesa/ | CCVCV | 2 | CCV.CV |
| 26 | pan | bread | /ˈpan/ | CVC | 1 | CVC |
| 27 | sed | thirsty | /ˈsed̪/ | CV(C) | 1 | CV(C) |
| 28 | leche | milk | /ˈleʧe/ | CVCV | 2 | CV.CV |
| 29 | chocolate | chocolate | /ʧokoˈlat̪e/ | CVCVCVCV | 4 | CV.CV.CV.CV |
| 30 | chicle | gum | /ˈʧikle/ | CVCCV | 2 | CV.CCV |
| 31 | flor | flower | /ˈfloɾ/ | CCVC | 1 | CCVC |
| 32 | llueve | It's raining | /ˈʤweβe/ | CVVCV | 2 | CVV.CV |
| 33 | nieve | snow | /ˈnjeβe/ | CVVCV | 2 | CVV.CV |
| 34 | primavera | spring | /pɾimaˈβeɾa/ | CCVCVCVCV | 4 | CCV.CV.CV.CV |
| 35 | elefante | elephant | /eleˈfant̪e/ | VCVCVCCV | 4 | V.CV.CVC.CV |
| 36 | cocodrilo | crocodile | /kokoˈd̪ɾilo/ | CVCVCCVCV | 4 | CV.CV.CCV.CV |
| 37 | jirafa | giraffe | /xiˈɾafa/ | CVCVCV | 3 | CV.CV.CV |
| 38 | pájaro | bird | /ˈpaxaɾo/ | CVCVCV | 3 | CV.CV.CV |
| 39 | pluma | feather | /ˈpluma/ | CCVCV | 2 | CCV.CV |
| 40 | jaula | cage | /ˈxau̯la/ | CVVCV | 2 | CVV.CV |
| 41 | hipopótamo | hippopotamus | /ipoˈpot̪amo/ | VCVCVCVCV | 5 | V.CV.CV.CV.CV |
| 42 | grande | big | /ˈgɾand̪e/ | CCVCCV | 2 | CCVC.CV |
| 43 | dinosaurio | dinosaur | /d̪inoˈsau̯ɾjo/ | CVCVCVVCVV | 4 | CV.CV.CVV.CVV |
| 44 | conejo | rabbit | /raˈt̪on/ | CVCVCV | 3 | CV.CV.CV |
| 45 | ratón | rmouse | /koˈnexo/ | CVCVC | 2 | CV.CVC |
| 46 | zanahoria | carrot | /sanaˈoɾja/ | CVCVVCVV | 4 | CV.CV.V.CVV |
| 47 | caballo | horse | /kaˈβaʝo/ | CVCVCV | 3 | CV.CV.CV |
| 48 | estanque | pond | /esˈt̪aŋke/ | V(C)CVCCV | 3 | V(C).CVC.CV |
| 49 | tortuga | turtle | /t̪oɾˈt̪uɣa/ | CVCCVCV | 3 | CVC.CV.CV |
| 50 | papá | papa | /paˈpa/ | CVCV | 2 | CV.CV |
| 51 | hermano | brother | /eɾˈmano/ | VCCVCV | 3 | VC.CV.CV |
| 52 | familia | family | /faˈmilja/ | CVCVCVV | 3 | CV.CV.CVV |
| 53 | muñeca | doll | /muˈɲeka/ | CVCVCV | 3 | CV.CV.CV |
| 54 | boca | mouth | /ˈboka/ | CVCV | 2 | CV.CV |
| 55 | nariz | nose | /naˈɾi{s}/ | CVCV(C) | 2 | CV.CV(C) |
| 56 | brazo | arm | /ˈbɾaso/ | CCVCV | 2 | CCV.CV |
| 57 | pierna | leg | /ˈpjeɾna/ | CVVCCV | 2 | CVVC.CV |
| 58 | bailando | dancing | /bai̯ˈland̪o/ | CVVCVCCV | 3 | CVV.CVC.CV |
| 59 | oigo | I hear | /ˈoi̯ɣo/ | VVCV | 2 | VV.CV |
| 60 | pelo | hair | /ˈpelo/ | CVCV | 2 | CV.CV |
| 61 | peine | comb | /ˈpei̯ne/ | CVVCV | 2 | CVV.CV |
| 62 | gorra | hat | /ˈgora/ | CVCV | 2 | CV.CV |
| 63 | pantalón | pants | /pant̪aˈlon/ | CVCCVCVC | 3 | CVC.CV.CVC |
| 64 | zapato(s) | shoe | /saˈpat̪os/ | CVCVCV(C) | 3 | CV.CV.CV(C) |
| 65 | guante(s) | glove | /ˈgwant̪e{s}/ | CVVCCV(C) | 2 | CVVC.CV(C) |
| 66 | reloj | watch/clock | /reˈlox/ | CVCVC | 2 | CV.CVC |
| 67 | sombrero | hat | /somˈbɾeɾo/ | CVCCCVCV | 3 | CVC.CCV.CV |
| 68 | guitarra | guitar | /giˈt̪ara/ | CVCVCV | 3 | CV.CV.CV |
| 69 | toca | she plays | /ˈt̪oka/ | CVCV | 2 | CV.CV |
| 70 | cámara | camera | /ˈkamaɾa/ | CVCVCV | 3 | CV.CV.CV |
| 71 | foto | photo | /ˈfot̪o/ | CVCV | 2 | CV.CV |
| 72 | juguete(s) | toy | /xuˈɣet̪es/ | CVCVCV(C) | 3 | CV.CV.CV(C) |
| 73 | globo(s) | balloon | /ˈgloβos/ | CCVCV(C) | 2 | CCV.CV(C) |
| 74 | bloque(s) | blocks | /ˈblokes/ | CCVCV(C) | 2 | CCV.CV(C) |
| 75 | regalo | present | /reˈɣalo/ | CVCVCV | 3 | CV.CV.CV |
| 76 | gracia(s) | thank you | /ˈgɾasja{s}/ | CCVCVV(C) | 2 | CCV.CVV(C) |
| 77 | saltando | jumping | /salˈt̪and̪o/ | CVCCVCCV | 3 | CVC.CVC.CV |
| 78 | martillo | hammer | /maɾˈt̪iʝo/ | CVCCVCV | 3 | CVC.CV.CV |
| 79 | clavo(s) | nail | /ˈklaβo/ | CCVCV(C) | 2 | CCV.CV(C) |
| 80 | tres | three | /ˈt̪ɾe{s}/ | CCV(C) | 1 | CCV(C) |
| 81 | lápiz | pencil | /ˈlapi{s}/ | CVCV(C) | 2 | CV.CV(C) |
| 82 | dragón | dragon | /d̪ɾaˈɣon/ | CCVCVC | 2 | CCV.CVC |
| 83 | dos | two | /ˈd̪o{s}/ | CV(C) | 1 | CV(C) |
| 84 | princesa | pirncess | /pɾinˈsesa/ | CCVCCVCV | 3 | CCVC.CV.CV |
| 85 | cuatro | four | /ˈkwat̪ɾo/ | CVVCCV | 2 | CVV.CCV |
| 86 | bruja | witch | /ˈbɾuxa/ | CCVCV | 2 | CCV.CV |
| 87 | rojo | red | /ˈroxo/ | CVCV | 2 | CV.CV |
| 88 | cruz | cross | /ˈkɾu{s}/ | CCV(C) | 1 | CCV(C) |
| 89 | flecha | arrow | /ˈfleʧa/ | CCVCV | 2 | CCV.CV |
| 90 | chocando | crashing | /ʧoˈkand̪o/ | CVCVCCV | 3 | CV.CVC.CV |
| 91 | ruido | noise | /ˈrwiðo/ | CVVCV | 2 | CVV.CV |
| 92 | llorando | crying/ | /ʤoˈɾand̪o/ | CVCVCCV | 3 | CV.CVC.CV |
| 93 | hoy | today | /ˈoi̯/ | VV | 1 | VV |
| 94 | playa | beach | /ˈplaʝa/ | CCVCV | 2 | CCV.CV |
| 95 | noche | night | /ˈnoʧe/ | CVCV | 2 | CV.CV |
| 96 | día | day | /ˈd̪ia/ | CVV | 2 | CV.V |
| 97 | agua | water | /ˈaɣwa/ | VCVV | 2 | V.CVV |
| 98 | barco | ship | /ˈbaɾko/ | CVCCV | 2 | CVC.CV |
| 99 | veinte | twenty | /ˈbei̯nt̪e/ | CVVCCV | 2 | CVVC.CV |
| 100 | Paula | euro | /ˈpau̯la/ | CVVCV | 2 | CVV.CV |
