# Supplementary material for: Development of word and syllable structure in Chilean children with typical and protracted phonological development
Source: Front Psychol. 2026 Mar 16;17:1740877. doi: 10.3389/fpsyg.2026.1740877 (PMC13033755; doi:10.3389/fpsyg.2026.1740877)
Supplement: Supplementary file 2 [file Table_2.docx]

Supplementary Material

**Supplementary Table 2.**

**Table S2***Descriptive statistics (mean and SD) for Whole Word Match, Percentage of Consonants Correct, and CV Match by age group and developmental profile (TD and PPD)*

| Age range | TD  WWM % Mean (SD) | PCC % Mean (SD) | CV Match % Mean (SD) | PPD  WWM % Mean (SD) | PCC % Mean (SD) | CV Match % Mean (SD) |
| --- | --- | --- | --- | --- | --- | --- |
| 3;0–3;5 | 56 (18) | 82 (5) | 88 (4) | 19 (7) | 51 (11) | 73 (6) |
| 3;6–3;11 | 71 (18) | 88 (8) | 93 (6) | 26 (17) | 56 (21) | 77 (10) |
| 4;0–4;5 | 74 (11) | 90 (4) | 93 (4) | 32 (16) | 66 (12) | 78 (7) |
| 4;6–4;11 | 89 (7) | 96 (3) | 98 (1) | 33 (22) | 63 (18) | 79 (11) |
| 5;0–5;5 | 93 (6) | 97 (2) | 99 (1) | 50 (22) | 76 (17) | 85 (11) |
| 5;6–5;11 | 93 (7) | 98 (3) | 98 (3) | 44 (19) | 73 (14) | 85 (10) |
| 6;0–6;5 | 95 (3) | 98 (1) | 99 (1) | 53 (22) | 79 (11) | 87 (7) |
| 6;6–6;11 | 96 (2) | 99 (1) | 99 (0) | 68 (13) | 87 (6) | 91 (4) |

*Note.* TD = typical development; PPD = protracted phonological development. WWM = Whole Word Match; PCC = Percentage of Consonants Correct; CV Match = proportion of consonant–vowel structures correctly produced. Values are means with standard deviations in parentheses. Each age band includes *n* = 10 children per group.
